# Case report: Progressive pulmonary artery hypertension in a case of megalencephaly-capillary malformation syndrome

**DOI:** 10.3389/fgene.2023.1221745

**Published:** 2023-08-08

**Authors:** Yuri Yoh, Tadashi Shiohama, Tomoko Uchida, Ryota Ebata, Hironobu Kobayashi, Kentaro Okunushi, Mitsuhiro Kato, Kazuki Watanabe, Mitsuko Nakashima, Hirotomo Saitsu, Hiromichi Hamada

**Affiliations:** ^1^ Department of Pediatrics, Chiba University Hospital, Chiba, Japan; ^2^ Department of Pediatrics, Showa University School of Medicine, Tokyo, Japan; ^3^ Department of Biochemistry, Hamamatsu University School of Medicine, Hamamatsu, Japan

**Keywords:** megalencephaly-capillary malformation syndrome, overgrowth syndrome, pulmonary arterial hypertension, PIK3CA, PI3K/Akt/mTOR pathway

## Abstract

Megalencephaly-capillary malformation syndrome (MCAP, OMIM # 602501) is caused by hyperactivity of the thephosphoinositide-3-kinase (PI3K)–Vakt murine thymoma viral oncogene homolog (AKT)–mammalian target of rapamycin (mTOR) pathway, which results in megalencephaly, capillary malformations, asymmetrical overgrowth, and connective tissue dysplasia. Herein, we report the case of a 7-month-old girl with MCAP due to a *PIK3CA* somatic mosaic variant who presented with atrial tachycardia, finally diagnosed as pulmonary arterial hypertension (PAH). Oxygen therapy and sildenafil decreased pulmonary blood pressure and improved atrial tachycardia. Previous studies reported an association between the PI3K/AKT/mTOR pathway and abnormal pulmonary arterial smooth muscle cell proliferation, which may be associated with PAH. PAH should be considered a potentially lethal complication in MCAP patients, even when no structural cardiac abnormalities are identified in the neonatal period.

## 1 Introduction

Megalencephaly-capillary malformation syndrome (MCAP, OMIM # 602501) is an overgrowth disorder characterized by a highly recognizable constellation of features involving the brain and body, including megalencephaly or hemimegalencephaly with perisylvian polymicrogyria, focal or generalized somatic overgrowth, vascular malformations, particularly capillary malformations, digital anomalies, variable connective tissue dysplasia, hypotonia, and mild-to-severe intellectual disability (Mirzaa G. M. et al., 2013). A heterozygous pathogenic variant of the *PIK3CA* gene has been identified in MCAP, and variants in this gene activate the thephosphoinositide-3-kinase (PI3K)–Vakt murine thymoma viral oncogene homolog (AKT)–mammalian target of rapamycin (mTOR) pathway, which leads to excessive growth in affected tissues (Riviére et al., 2012).

As the symptoms and severity of MCAP vary greatly from one person to another, their life expectancy is also variable. Sudden infant death has been reported in at least 12 children with MCAP (Yano and Watanabe, 2001; Nakamura et al., 2014; reviewed by Harada et al., 2015). The causes of death include heart failure due to congenital heart disease, arrhythmia, and cerebellar tonsillar herniation; however, in many cases, the reasons remain unclear (Harada et al., 2015).

Herein, we report a patient with MCAP with a somatic heterozygous pathogenic variant of *PIK3CA* who presented with atrial tachycardia and progressive pulmonary arterial hypertension (PAH). Non-structural cardiovascular abnormalities in MCAP have not been reported in detail (Mirzaa G. et al., 2013; Garde et al., 2021), except for the patients with structural heart defects (e.g., atrial and ventricular septal defects) and/or abnormalities of the great vessels (Yano and Watanabe, 2001; Mirzaa G. et al., 2013; Mirzaa et al., 2016), the clinical course in our case may shed light on PAH as a potential life-threatening comorbidity.

## 2 Case presentation

**FIGURE 1:**
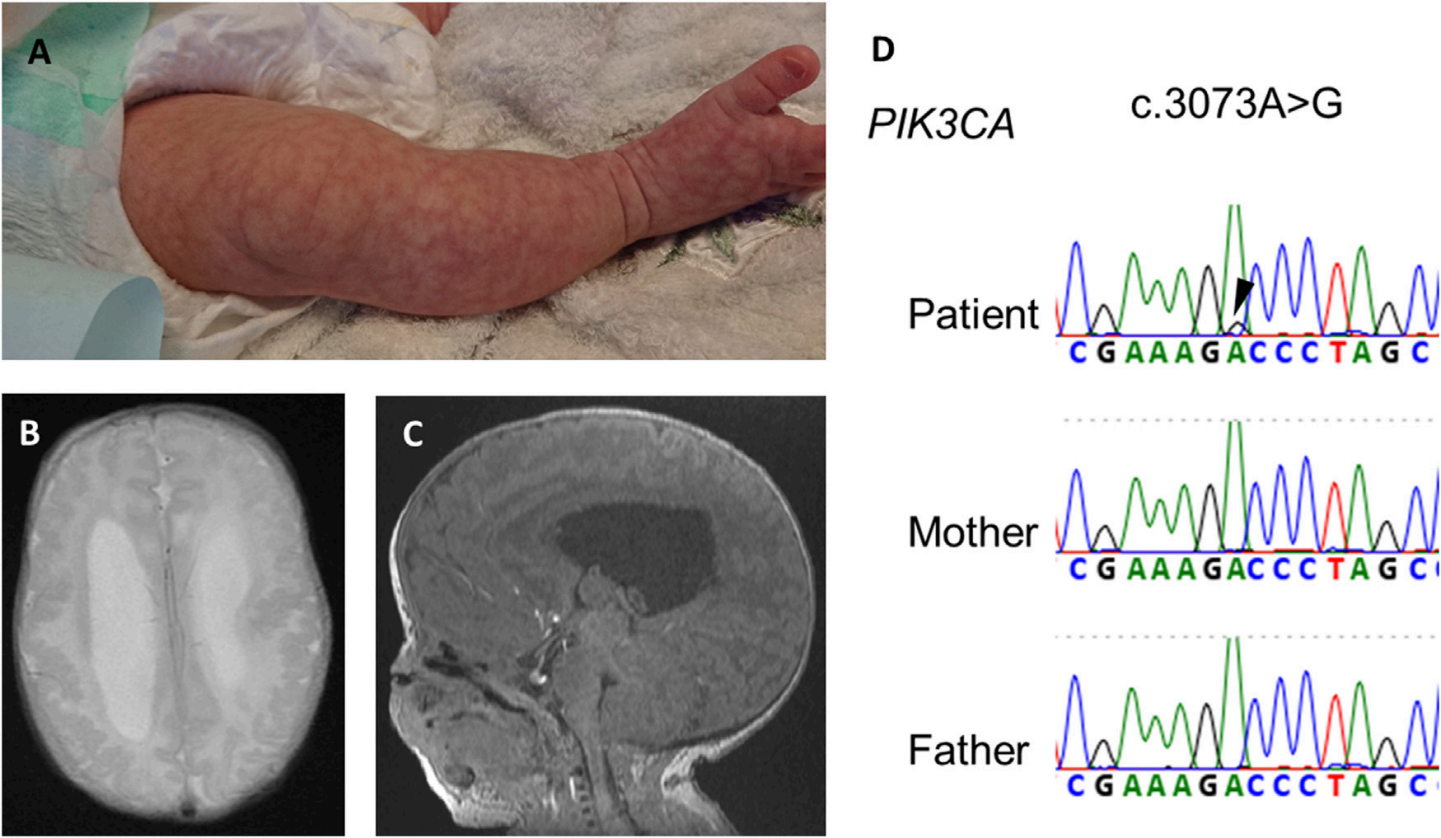
Physical and neuroimaging findings at the neonatal period. Cutis marmorata and sandal gap toe in the right leg **(A)**, extensive bilateral polymicrogyria and ventricular dilatation [**(B)**, axial t2-weighted image], and mild sagging of the central cerebellum [**(C)**, sagittal T1-weighted image]. **(D)** Electropherogram of Sanger sequencing of the patient and his parents. Black arrowheads indicates a somatic mosaic *PIK3CA* variant.

A girl was referred at 22 weeks of pregnancy because due to large for her gestational age with an estimated weight of 1,072 g (+4.4 SD). She also showed enlarged lateral ventricles and excessive amniotic fluid. She had no family history of genetic disorders or PAH. She was born by cesarean section at 35 weeks and 5 days of age, with a birth weight of 4,391 g (+5.4 SD), height of 52 cm (+3.2 SD), and occipitofrontal circumference of 44 cm (+9.6 SD). She showed macrocephaly, neonatal hypotonia, frontal bossing, cutis marmorata (Figure 1A), hyperextensibility of the lateral abdomen, syndactyly and sandal gap toe on both feet, and polydactyly of the right hand. Brain magnetic resonance imaging on 18 days of age revealed extensive polymicrogyria (Figure 1B) and mild sagging of the central cerebellum (Figure 1C). Her physical and neuroimaging findings were compatible with MCAP.

At 2 months of age, she presented with hypoglycemia due to low serum cortisol. She was diagnosed with adrenal hormone deficiency and was treated with hydrocortisone. In addition, tracheostomy and ventriculoperitoneal shunt were performed for laryngomalacia and hydrocephalus, respectively, at 3 months of age.

**FIGURE 2:**
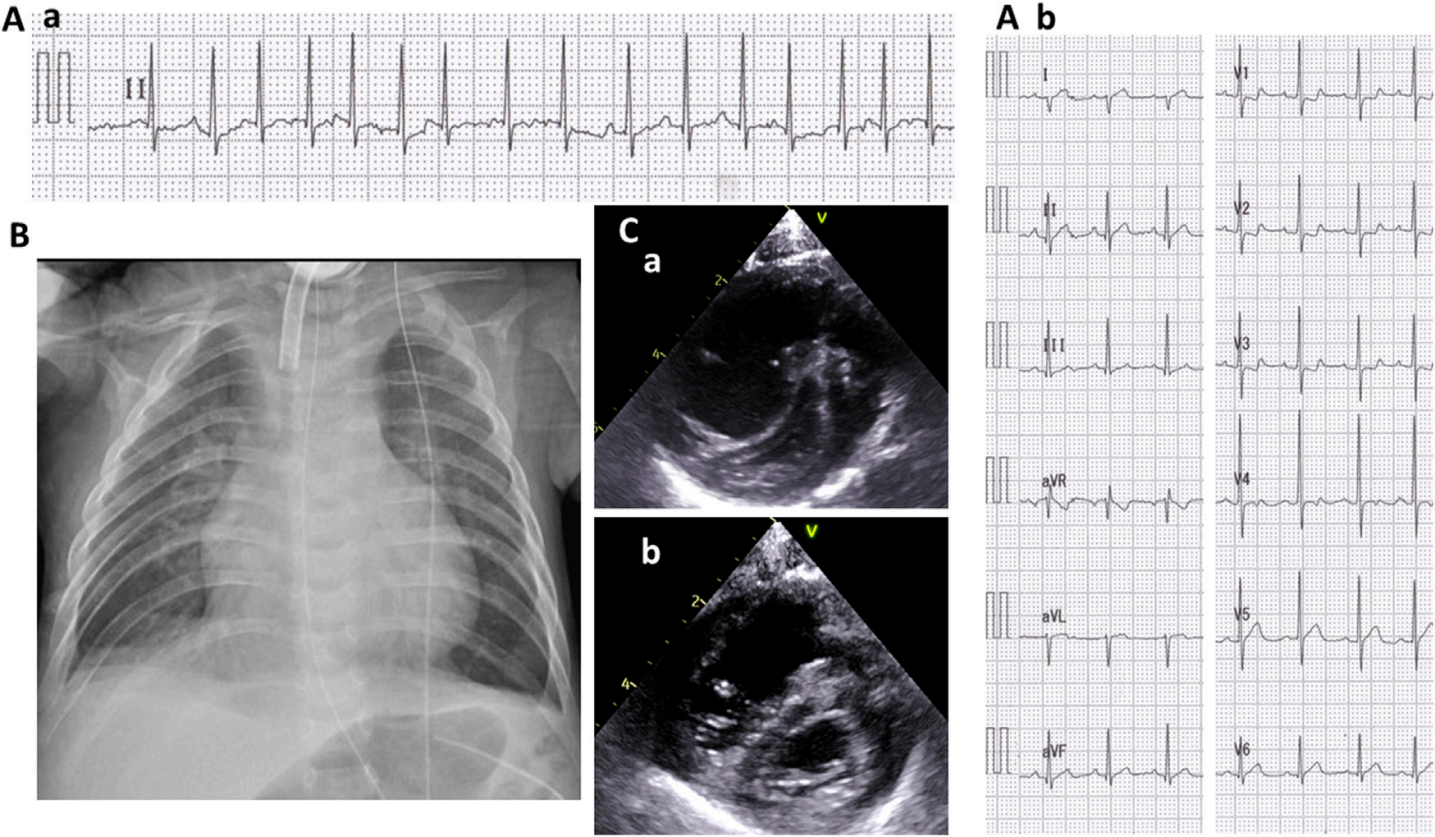
Cardiologic evaluation at 7 months old. A II-lead electrocardiogram (ECG) **(A-a)** when presenting with tachycardia and respiratory distress and a 12-lead ECG **(A-b)** after recovering sinus rhythm. A chest radiograph showing cardiomegaly, pulmonary congestion, and consolidation **(B)**. A short-axis view of echocardiography when presenting tachycardia and respiratory distress **(C-a)** and after the initiation of treatment for pulmonary hypertension **(C-b)**.

At the age of 5 months, she suddenly developed tachycardia of 190–200 beats/min due to ectopic atrial tachycardia. Administrations of digoxin improved her cardiac rhythm, then returned to sinus rhythm. At 7 months of age, she exhibited labored respiration, tachycardia (200–220/min), hypoxia (SpO_2_ 88%), and an accentuated second heart sound. Serum brain natriuretic peptide levels were 722 pg/ml, which suggested cardiac failure. The blood digoxin concentration was within the appropriate range (1.2 ng/ml). Chest X-ray showed cardiomegaly, pulmonary congestion, and consolidation (Figure 2B). Electrocardiogram revealed narrow QRS tachycardia with irregular RR intervals (Figure 2A-a). Echocardiography revealed D-shaped left ventricle (Figure 2C-a) and tricuspid regurgitation, and the estimated right ventricular pressure by the tricuspid regurgitation pressure gradient was 96 mmHg, while her systolic blood pressure was 84 mmHg. Pulmonary vein stenosis was not observed, nor was mitral valve or aortic stenosis. Echocardiography and contrast enhanced computed tomography (CT) revealed no portal vein abnormalities.

She was diagnosed with PAH and treated with rest and oxygen (FiO_2_ 0.5). Her rhythm returned to the sinus rhythm (Figure 2A-b), and her heart rate reduced to 130–140/min. The shift of the ventricular septum improved (Figure 2C-b), and the estimated right ventricular pressure decreased to 50 mmHg.

Blood tests revealed normal routine biochemistry, hematology, negative antinuclear antibodies, normal total bile acid (8.7 μmol/l), and high KL-6 (567 U/ml). Echocardiography revealed a small atrial septal defect but no other cardiac malformations, such as pulmonary vein stenosis or left-sided heart diseases. Chest CT revealed atelectasis and inflammatory findings, but chronic lung disease and interstitial pneumonia were not suggested. Pulmonary venous-occlusive disease, chronic thromboembolic PAH, bronchopulmonary hypoplasia, and pulmonary artery hypoplasia were not observed. Taken together, these results suggest that her PH was due to PAH.

In addition to oxygen, sildenafil was administered, and her condition improved. We adjusted the ventilator FiO_2_ to 0.3, PIP/PEEP to 15/6 cmH2O, and estimated that the right ventricular pressure decreased to 55 mmHg on echocardiography. We increased sildenafil to 2.0 mg/kg/day eventually. She was weaned from mechanical ventilator at 14 months of age, while PAH persisted to require additional treatment with phosphodiesterase-5 inhibitor at 16 months of age.

After obtaining informed consent, we performed exome sequencing (xGen Exome Research Panel, Integrated DNA Technologies) for the proband using DNA extracted from blood and identified a somatic mosaic *PIK3CA* variant (NM_0062184.4: c.3073A>G, p.(Thr1025Ala)) in the patient. The variant allele fraction (VAF) of this variant was 9% (4/46, variant reads per total reads). The variant from exome sequencing was confirmed by Sanger sequencing (Figure 1D). The variant protein site of this case is located near His1047 within the kinase domain of PI3KCA. The kinase domain mutation His1047Arg induces a conformational change independently of RAS-binding to enhance PI3K/AKT/mTOR signaling (Zhao and Vogt, 2008).

We confirmed the VAF by deep sequencing, as described previously (Nakashima et al., 2014). Genomic DNA derived from blood leukocytes was PCR-amplified using the following primers: forward 5′-TCT​AGC​TTA​AGC​ACA​AAA​GGA​TTT-3′ and reverse 5′-CAA​TCT​TCA​AAG​TTT​ACC​TTT​TTG​G-3′. A single-indexed sequencing library was prepared using the NEBNext Ultra II FS DNA Library Prep Kit for Illumina (NEW ENGLAND Bio Labs, Ipswich, MA) and sequenced on an Illumina MiSeq (Illumina, San Diego, CA) with 150-bp paired-end reads. Quality-filtered reads were mapped to the human reference genome sequence (GRCh 38) and aligned using BWA-MEM (Version 0.7.17), and the aligned read files in the BAM format were sorted and indexed using SAMtools. Data analysis, including allele counting, was performed by Integrative Genomics Viewer software. The deep sequencing revealed that the VAF of this variant was 9.17% (805/8771, variant reads per total reads).

This somatic variant was not registered in the genome aggregation database (http://gnomad.broadinstitute.org/) and was predicted to be deleterious by multiple *in silico* pathogenicity prediction tools. The same variant has been identified in one case among 24 cases of MCAP with *PIK3CA* variants (Mirzaa G. M. et al., 2013), suggesting this somatic variant is causative for MCAP in this patient; however, there was no clinical information including the presence of PAH in the previous literature. Additionally, other case series of MCAP with *PI3KCA* variants identified no patient with PAH (Garde et al., 2021).

## 3 Discussion

The current case of MCAP presented with atrial tachycardia and severe PAH. PAH persisted even after weaning from respiratory support. Taken together with the 12-lead electrocardiogram findings, the preceding presence of atrial load findings due to elevated right ventricular pressure, absence of tachycardia during the neonatal period when tachycardia is more likely to occur, and resolving of tachycardia with improvement of transiently exacerbated PAH, we concluded the diagnosis was atrial tachycardia due to PAH. MCAP is an entity within the clinical spectrum of megalencephaly and overgrowth syndromes caused by constitutive hyperactivation of PI3K/AKT/mTOR signaling, which plays a crucial role in numerous cellular functions, including cell growth, proliferation, survival, migration, metabolism, angiogenesis, apoptosis, and brain development (Riviére et al., 2012).

PI3Ks are heterodimeric enzymes that convert phosphatidylinositol-4,5-bisphosphate (PIP2) to phosphatidylinositol-3,4,5-trisphosphate (PIP3), which interacts with AKT at the cellular membrane, where it is phosphorylated and activated. An important negative regulator of PI3K is tumor suppressor phosphatase and tensin homolog (PTEN), which dephosphorylates PIP3. AKT modulates a range of cellular metabolic and cell survival functions through indirect activation of mTOR, which controls cell proliferation, protein translation, and autophagy (Riviére et al., 2012; Weigelt and Downward, 2012).

*PIK3CA* variants associated with MCAP have been reported to cause PI3K hyperactivity and PI3K/AKT/mTOR signaling in cells derived from affected individuals (Loconte et al., 2015; Suzuki et al., 2017; Ranieri et al., 2018). Although in the current case, somatic mosaic expression of a pathogenic *PIK3CA* variant was identified in lymphocytes, various types of cells with increased PI3K/AKT/MTOR signaling may be distributed in various organs.

PAH is a progressive and fatal disease characterized by pulmonary vascular resistance and pulmonary arterial pressure (Morrell et al., 2009; Babicheva et al., 2021). PAH progression is related to pulmonary vasoconstriction, concentric pulmonary vascular remodeling, local thrombosis, and pulmonary artery wall thickening (Galiè et al., 2009; Babicheva et al., 2021). Wall thickening is driven by increased proliferation and inhibited apoptosis among pulmonary vascular endothelial cells (EC), pulmonary smooth muscle cells (PASMC), and fibroblasts (FB) (Maron et al., 2020; Ratwatte et al., 2020; Babicheva et al., 2021). Interestingly, the molecular basis of pulmonary artery wall thickening is closely related to PI3K/AKT/mTOR signaling (reviewed by Babicheva et al., 2021). PI3K functions downstream of BMPR2, a gene associated with hereditary PAH (Deng et al., 2000; Andruska and Spiekerkoetter, 2018). Although the genetic background for PAH has not yet been elucidated, *BMPR2*, *KCNK3*, *ABCC8*, *TBX4*, and *SOX17* were known as familial PAH-related genes (Southgate et al., 2020). At least, no causative variant in these five genes was identified in the current case.

In cellular models of PAH, proliferation, and migration of pulmonary artery muscle cells were accelerated through AKT phosphorylation. The rescue could be achieved with PI3K inhibitors (ten Freyhaus et al., 2011). Several PI3K/AKT/MTOR signaling inhibitors have been tested in clinical trials for the treatment of PAH (reviewed by Babicheva et al., 2021). We also have a strong interest if PIK3CA inhibitor or mTOR inhibitor effectively works for her PAH; however, we could not use them since they were not approved by Pharmaceuticals and Medical Devices Agency in Japan.

On the other hand, the frequency of PAH in MCAP patients remains unclear. Among 75 MCAP patients, four children presented with cardiac arrhythmia and died suddenly. PAH was not mentioned in these cases (Lapunzina et al., 2004). It is not clear why PAH is uncommon in MCAP, but it may be associated with variability in somatic mosaic tissue distribution as a feature of MCAP. There may be other cases of PAH that are milder and not identified. We did not directly prove that PAH in her was caused by the *PIK3CA* variant because we could not obtain any biopsy sample of the proliferative tissue from her lung. Analysis of lung tissue at autopsy in multiple cases of MCAP with *PI3KCA* variants would be necessary to prove causality.

Some congenital disorders, including Down syndrome (DS), are at a greater risk of PAH than the general population, partly due to upper airway obstruction and congenital heart disease (Hawkins et al., 2011; Pandit and Fitzgerald, 2012). Indeed, the frequency of early progression of histopathological pulmonary arteriopathy did not differ between patients with and without DS (Masaki et al., 2018). In contrast, the current patient had already undergone tracheostomy at 2 months of age and had a stable airway without hypoxia or hypercapnia for more than 3 months. In addition, apparent lung damage (e.g., pneumonia, atelectasis, or emphysema) was not identified.

## 4 Conclusion

We have reported the case of an MCAP patient with a somatic *PIK3CA* variant, whose condition was complicated by severe PAH. Although PAH in MCAP is a rare complication, we should pay attention to cardiological findings, including the atrial load findings due to elevated right ventricular pressure which could be screened by chest radiography and electrocardiogram in patients with MCAP.

## Data Availability

The original contributions presented in the study are included in the article/Supplementary Material, further inquiries can be directed to the corresponding author.
